# 

**Published:** 1889-08-17

**Authors:** 


					310 THE HOSPITAL, August 17, 1889.
SPECIAL ANNOUNCEMENT.
MISS FLORENCE NIGHTINGALE.
The next (September) monthly part of The Hospital will be accom-
panied by a portrait Of Miss Nightingale, which has been reproduced
at great cost and perfection ; and it will at last be possible for every
member of the nursing profession to possess the likeness of one who,
by a life of self-sacritice and philanthropic labour, lias raised herself
to pre-eminence among women. The monthly number will be issued at
sixpence as usual, but orders should be sent early.
We regret that, owing to an accident which has happened to the
plate at the collotyping works where this portrait is being produced,
we were compelled to postpone its publication to the next (Sep-
tember) monthly part. Though the delay thus caused is much to be
regretted, there is consolation in the knowledge that a really excellent
portrait of Miss Nightingale will be within reach of our readers a few
weeks hence. The biographical sketch appeared in our issue of July 27.
NOTICE TO CORRESPONDENTS.
AH MS., Letters, Books for Review, and other matters intended for the
Editor, should be addressed The Editor, The Lodge, Porchester
Square, London, "W.
Notice to Advertisers and Subscribers.
All Advertisements, Orders for Copies of The Hospital, and Business
Communications should be addressed to The Publisher, not to the Editor,
The Hospital, 139-140, Salisbury-court, Fleet-street, E.C.
Vol. V., now ready, 4s., post free. Cases for binding, may be had of
the Publisher, at Is. 6d. each.
To Residents, Secretaries, and Matrons.
The Editor will be glad to have the Regulations and each Report as
issued by Asylums, Hospitals, Convalescent and Nursing Institutions, as
well as those of other Charities, and an early copy in duplicate of all
Appeals and Festival Notices, etc., addressed to The Editor, The Lodge,
Porchestersquare, London, W. Any superintendent, secretary, matron,
or other chief official who regularly sends such information may be
registered, on application to the Publisher, to receive free of cost a cover
er binding The Hospital in half-yearly volumes.
The Students Column.
UNIVERSITY OF LONDON.
EXAMINATION FOR HONOURS.
Anatomy.?First-class?Wm. M. Stevens (Exhibition and Gold Medal),
Diversity College. Second-class?Alfred Theodore Rake, Guy's Hos-
pital; Charles Henry Preston, Owens College and Manchester Royal
nflrniary. Third-class?William Lloyd Andriezen, University College ;
Barnett Warrington, Owens College ; Edward Chichester, London
osPital; Thomas Holmes, Guy's Hospital.
Physiology and Histology.?First-class?Wm. M. Stevens (Exhibi-
'01> and Gold Medal), University College; Harold M. Richards (Gold
I. e. a')> University Jollege. Second-class?William Lloyd Andriezen,
j 'lu ei'sity College. Third-class?Alfred Theodore Rake, Guy's Hospital;
^ meg Edward Ramsay, University College; Edward Thomas Ernest
Ho Guy's Hospital; George Lancelot Rolleston, St. Bartholomew's
spital and Cambridge University: Emily Elizabeth Wood, London
Sch?ol of Medicine for Women.
^0j''Ganic^Chemistry.-First-class? William Lloyd Andriezen (Exhibi-
jtj ^ an^ Gold Medal), University College; George Seaton Buchanan,
Hospit ]' ^ar^'?^oniew s Hospital; Edward Eldridge Blomfield, Loudon
WurTEIUA ^lEI)1CA and Pharmaceutical Chemistry.?First-class?
Tho,lam Stevens (Exhibition and Gold Medal), University College ;
Collet ^?^mes> Guy's Hospital; William Lloyd Andriezen, University
firnia"6- ^ucon<l-class?John C. Mackmurdo Given, Liverpool Royal In-
Edw l\" ^hird-class?Arthur Man tell Daldy, Guy's Hospital; James
Vei'sity mSa^' University College > Harold Meredith Richards, Uni-
PASS LIST.
for w T ^>IVISION-~I'Ouisa B. Aldrich-Blake, London School of Medicine
iUau g U'?n ' Hugh Wells Armstead, St. Bartholomew's Hospital; Ches-
versitv p 6r' Sheffield Medical Institution; Vivian Boase Bennett, Uni-
Boscit ,ollege' Liverpool; Arthur Seal Blackwell, St. Bartholomew's
Cle-?r ^ ' Claude Churchill Chidell, University College; John Gray
Royal' TT,fie"s College; Richard Clegg, Owens College and Manchester
Owen r,;i"rrnary ? Charles Coles, St. Bartholomew's Hospital; Howard
Navies rT,le.S' Bartholomew's Hospital; Thomas Benjamin Philip
Charles Hospital; Charles Arthur Dixon, The Yorkshire College;
1'homas's TT - Elliott, Guy's Hospital; John Herbert Fisher, St.
Harvev V .P1^; Henry Wilkes Gibson, Middlesex Hospital; John
School 'of ?ilvf!s?ty College, Liverpool; Jessie Flewitt Hatch, London
Percy Mmiti cine for Women ; Percy Lord, Guy's Hospital; William
joinery, Owens College; Edward Albert Nathan, St. Mary's
Hospital; William Prior Purvis, St. Thomas's Hospital ; Herbert .Barn-
well Rowbotham, Queen's College, Birmingham; William Edward
Sargant, St. Bartholomew's Hospital; Alfred William Sheen, Guy's
Hospital; Gerald Allpress Simmons, St. Mary's Hospital; Hugh Roubiliae
Smith, University College; William Robert Smith, King's College;
Caroline Sturge, London School of Medicine for Women; John Odery
Symes, St. Mary's Hospital; Tom Robinson Taylor, Guy's Hospital;
William Longworth \\ ainwright, St. Thomas's Hospital; Cuthbert
Sidney Wallace, St. Thomas's Hospital; George Alexander Watson, Uni-
versity College; Arthur Nesham Weir, B.Sc., St. Bartholomew's Hos-
pital ; Charles Edwin Wheeler, St. Bartholomew's Hospital; Arthur
Whitfield, King's College; Samuel Williams, University College;
Charles Jerome Woollett, Charing Cross Hospital.
SECOND Division.?Tom William Beazeley, Queen's College, Birming-
ham ; Charles Bernard Braithwaite, Guy's Hospital; Stanley Mewburn
Brown, Owens College; Frank P. S. Cresswell, B.Sc., Guy's Hospital;
Howard Edward Girdlestone, St. Thomas's Hospital; Edgar Huntley,
Guy's Hospital; Thomas David Lister, Guy's Hospital; James Hannay
Muncaster, Guy's Hospital; Alonzo George Rider, University College;
Mary Louisa Sprigg, London School of Medicine for Women; George
Robert Fabris Stillwell, St. Thomas's Hospital; Edward Gaved Stocker,
St. Bartholomew's Hospital; Wilfrid Watkins-Pitchford, St. Thomas's
Hospital; Harry Bacon Wilkinson, Guy's Hospital; Alfred Field Henry
Wray, St. Bartholomew's Hospital ; Sydney Faulconer Wright, St.
Thomas's Hospital.
Excluding Physiology.
First Division.?Edward Buller Allan, University College ; William
Tillinghast Atwool, St. Mary's Hospital; James Benzeville Byles, Lni-
versity College ; Rowland N. U. Pickering, St. Bartholomew s Hospital.
Second Division.?Harry Finley, University College; Frank Drew-
Harris, St. Mary's Hospital; Alfred Hugh Minton, King s College;
George Dines Parker, St. Bartholomew's Hospital; Reginald Mander
Smyth, St. Mary's Hospital; James Hugh Sproat, Queen s College, Bir-
mingham ; Henry Eugene Tracey, St. Bartholomew s Hospital; Arthur
Longley Whitehead, Yorkshire College.
Physiology Only.
Second Division.?John James Brown, Guy's Hospital; Joseph
Richard Buckley, Owens College; Rupert James, University College;
Geoffrey Colley March, Owens College ; Elizabeth Margaret Pace, Lon-
don School of Medicine for Women ; Charles Barre Phipps Spencer,
Guy's Hospital; Mildred E. K. Staley, London School of Medicine for
Women; Frederick Wallace Wilson, Guy s_ Hospital; John Grattoii'
Wilson, London Hospital.
Amusements and Relaxation.
Second Quarterly Word Competition Commenced
July 6th, ends September 28th.
Ihree Prizes of 15s., 10s., 5s., will be given for the largest number of
-words derived from the words set for dissection.
Proper names, abbreviations, foreign words, words of less than four
letters, and repetitions are barre,d; plurals, and past and present par-
ticiples of verbs, are allowed. Nuttall's Standard dictionary only to be
used.
N.B.?Word dissections must be sent in WEEKLY, arranged alpha-
betically, with correct total affixed.
The word for dissection for this, the Seventh week of the quarter, being
"POTSDAM."
Results of Fifth Week.
Names. Aug. 8th.
Nurse Duty   37
N'importe Qui .. 34
Jumps  ?
Tinie   ?
Patience  42
Amicus   33
Broxbourne   34
Speedwell  32
, Sister   32
Lightowlers   42
Lauriston    42
Rattler   ?
Paignton   33
Fassifern   ?
W. R. E  37
Esperance  37
M. W  37
Isabel  37
Buxton   ?
Nux Vom  28
- Qu'appelle  33
Spes  ?
Secnarf   ?
Rover  ?
Names. Aug. 8tli
Leirion   ?
Matron   25
F. C. J  ?
Percy Verance .. ?
Gleniffer  ?
Gypsye   39.
Ladyr  ?
A. B  ?
Essie   29
W. G. R  31
Cowboy Bill  ?
M. L. A  22
Little Tuppy  ?
Embryo  ?
K. Jarman  ?
Reveal  ?
Jenny Wren .... 35
Condorcet  27
Electrician  36
Pearl   ?
R. W  _
Nurse Amie  ?
Sunshine  24
Notice to Correspondents.
Paignton, Fourth Week, 18.
N.B.?All letters referring to this page which do not arrive at 139 and
140. Salisbury-court, London, E.C., by the first post on Thursdays, and
are not addressed PRIZE EDITOR, will in future be disqualified and
- disregarded ?
Competitors can enter for all quarterly competitions, but no com-
?petitor may take more than t wo quarterly prizes during the year.
Conditions.
I. Every paper sent in must be clearly written, and one side only must
/be used. All competitors must mark at the head of their papers the
number of their competition.
II. Each paper must be signed by the author with his or her real name
and address. A norm de plume may be added if the writer does not desire
to be referred to by us by his real name. In the case of all prize-winners,
however, the real name and address will be published.
III. All communications relating to prize competitions should be ad-
dressed to "The Prize Editor, SHE HOSPITAL, 139 and 140, Salisbury-
court, London, E.C." Competitors are requested not to include in letters,
etc., to the Prize Editor communications on matters of other business.
All letters must be fully prepaid.
IV. The Prize Editor of THE HOSPITAL is the sole judge of the com-
petitions, and his deoision is in all cases final and without appeal.
- V. The Prize Editor reserves the right to publish any paper sent in,
whether it receives a prize or not. He does not hold himself responsible
tjoc any MSS. sent, nor can he undertake to return rejected competitions.
Scraps and Gleanings.
There ave 3,000 lepers on the Island of Crete.
Hereford kept Hospital Sunday on August 4.
Cholera still claims 400 victims a week in Can jam.
Dr. Weir Mitchell has published a volume of verse.
A NEW edition of "Sturges on Pneumonia " is announced.
A serious epidemic of smallpox has broken out at Jutland.
In Dispalla, Bengal, 150 deaths are reported from starvation.
Longton Cottage Hospital, recently opened, contains 28 beds.
Belfast Hospital Sunday has yielded ?300 against ?221 last year.
Kenilworth subscribed ?44 to Warneford Hospital on Hospital
Saturday.
Cholera of a virulent type has broken out in the Coimbatore, India,
central gaol.
Dr. G. R. Macgregor has been appointed medical officer of health to
Bingley district.
Newcastle Infirmary has received ?100, through Dr. Page, from an
unknown friend.
Dr. VV. K. Sebley has been appointed house physician at Brompton
Consumption Hospital.
North Staffordshire Infirmary receives ?200 by the will of the
late Mr. T. Hackwood.
Sheffield Infirmary and the Jessop Hospital both receive ?200 by
the will of the late Mr. J. H. Hunter.
The Queen has approved the bestowal of an Indian good-service
pension on Surgeon-General G. Bidie.
St. Mark's Ophthalmic Hospital has received ?104 6s. 8d., under
the will of the late Mrs. Selina Miller.
Victoria Park Hospital for Chest Diseases will in future be open for
the systematic instruction of students.
The British Dental Association will hold their annual meeting at
Brighton Pavilion on August 22, 23, and 24.
Miss F. Nightingale Toms, M.D., was married on July 25th, a'
Acton, to Mr. Stanley Boyd, M.B., F.R.C.S.
Dr. Alan Herbert, of Paris, has been feted by his many friends on
the occasion of his receiving the Legion of Honour.
Portsmouth Eye and Ear Hospital treated 12,700 patients in the
last six months. Funds are needed to carry on this work.
The Maidenhead Saturday collection, on behalf of the Cottage Hos-
pital, amounted to ?60 7s. 5d., or about ?15 more than last year.
The Empress of Japan lives very economically, and sends all her
savings to the Tokio Female Hospital. Has she no grandchildren ?
A considerable number of medical specialists from all parts of
Germany have attended the Austrio-German Anthropological Congress.
The first annual meeting of Carnarvon Cottage Hospital was held on
July 27th. The Mayor presided, and a satisfactory report was submitted.
Henry Hall, aged 13, died from hydrophobia last week in St. Mary's
Hospital. This is the second case of hydrophobia in Paddington in
three weeks.
Sir James Paget, Sir Henry Acland, Dr. Juain, Dr. Reid, and Dr.
Evans were amongst the medical celebrities present at the marriage oi
the Princess Louise of Wales.
According to the London Gazette, the number of dogs in England re-
ported, during the week ending July 27th, as afflicted with rabies was
seven, six of which were killed, whilst one died.
A crowded meeting was recently held at Sholapore to take measures
for endowing a medical school and hospital in connection with the Lady
Dufferin Fund. Miss Sorabjee gave an address.
A special meeting was held recently at the Animals' Institute to offer
the society medal and ?10 10s., given by Dr. Gale, the blind electrician,
for the best humane muzzle to prevent suffering to dogs. Entries close
on August 21st.
The girls of the West Dulwich High School have collected the sum
?100, which they have presented to the Children's Country Holidays
Fund; and by so doing have ensured a fortnight's holiday for at least
200 poor city arabs.
The summer session of the Army Medical School was brought to a
close on the 2nd instant at Netley. Ten candidates for commissions ?'
H.M.'s Indian Medical Service had passed through the courses of prac"
tical instruction at the school.
It is announced that the funds of the Welsh Calvinistic Methodist
Society have just been unexpectedly replenished by a munificent dona-
tion of ?100,000 from a Mrs. Roberts, a member of the Connexion, wn
has resided at Mold, Flintshire. ,
Through the kindness of Lady Radnor, better known in the East En
as Lady Folkestone, about fifty children from the neighbourhood o
Ste;mey have been enabled to spend a fortnight or three weeks on ti
L ?ngford Castle estate, near Salisbury.
The Memorial Convent to the unfortunate Crown Prince of Austria,
established in his shooting lodge at Meyerling, will be consecrated'
October, with great solemnity. The room where Prince Rudolph die
has already been converted into a chapel.
The vaccination officer reported to the Eastbourne Board of Guardians
that the unpopularity of vaccination in the Eastbourne district ?
growing rapidly. His return for the last six months showed that oi
births only 157 of the children had been vaccinated.
Warwick Hospital Saturday Fund has held its annual meeti'1^
After the payment of expenses, amountingto ?8 13s. 6d., the sum oi ?
had been devoted to the Warneford Hospital, ?1 19s. 9jd. to
Children's Ward, and ?26 to the Warwick Cottage Hospital.
A Deaf and Dumb Congress has just closed in Paris?the first SatAn.
ing of the kind ever held. The congress discussed the best means oi ^
structing deaf mutes, and also commemorated the centenary of tliei?,.ers
of the Abbe de l'Epee, who was the first person to instruct such suae
in France.

				

